# Evaluation of a new air water generator based on absorption and reverse osmosis

**DOI:** 10.1016/j.heliyon.2020.e05060

**Published:** 2020-09-25

**Authors:** Marc Fill, Flavio Muff, Mirko Kleingries

**Affiliations:** a Lucerne University of Applied Sciences and Arts, Institute of Mechanical Engineering and Energy Technology, Technikumstrasse 21, CH-6048 Horw, Switzerland; b Alera energies AG, Hohenrainstrasse 36, CH-6280 Hochdorf, Switzerland

**Keywords:** Chemical engineering, Mechanical engineering, Thermodynamics, Membrane, Transport process, Heat transfer, Mass transfer, Process Modelling, Absorption, Reverse osmosis, Air water generation, Modelling, Simulation

## Abstract

The evaluation of a new air water generator (AWG) based on absorption and reverse osmosis is described. For the evaluation, an aqueous lithium bromide solution has been selected from a wide range of liquids as the absorbent. At high salt mass fractions, the aqueous lithium bromide solution has a low vapour pressure and a high osmotic pressure. The low vapour pressure ensures that the water vapour can be absorbed from the air, but the high osmotic pressure leads to high pressures over the membrane. Due to the high osmotic pressures, several reverse osmosis membrane modules are necessary and salt solution has to be present on both sides of the membrane, which leads to an additional inlet on the permeate side. Models for the absorber, the reverse osmosis membrane module and the complete multi-stage reverse osmosis system have been developed in Python. The model of the complete system has then been used to simulate the performance of the AWG at different boundary conditions. The simulations have shown that based on the defined assumptions, extracting water from the air with absorption and reverse osmosis is possible and that the energy demand per litre of pure water is similar to AWG systems which use condensation.

## 1 Introduction

Water scarcity has been a global problem for many decades. Due to population growth and climate change, competition for access to fresh water is expected to increase further. Not only arid regions such as northern Africa or the Middle East are affected, but increasingly also European states such as Spain, Italy or even the west coast of the USA such as California [1], [2]. The World Health Organization and Unicef expect that India and China will also be increasingly affected by water crises on a large scale and that by 2025 around 1.8 billion people worldwide will suffer from a shortage of pure water [3]. Physically there is enough fresh liquid water on the earth's surface in the range of 90'000 km^3^ in the form of lakes and rivers for the supply of mankind [4]. In regions without sufficient resources of natural drinkable water, but access to sea water or polluted water, desalination or sewage treatment plants are suitable for the provision of clean water [5, p. 192]. If there is no such access to liquid water, water must be transported to the recipients. In many regions affected by water shortages, large facilities for the provision of sufficient quantities of clean water do not exist and permanent transport is not possible. Another possible source, which has not yet been truly exploited, is the water that is stored in the atmosphere. The earth's atmosphere contains such a large amount of water vapour that the liquid state of the water results in a volume of about 13'000 km^3^, which is about one seventh of the volume of fresh water on the earth's surface [4].

In order to extract water from the air, currently either the condensation of air humidity on cold surfaces with a cooling system or sorption with classical desorption (especially by temperature increase) is used. In the case of present seawater, reverse osmosis with membranes is used due to the high energy efficiency. In this paper the feasibility of a combination of absorption and reverse osmosis shall be investigated. With a hygroscopic aqueous solution, very probably a salt solution, water has first to be extracted from ambient air and with reverse osmosis instead of desorption the water has to be expulsed again.

## 2 Fundamentals

### 2.1 Osmosis

If two differently concentrated solutions are separated by a membrane which is only permeable to the solvent, osmosis occurs. The solvent attempts to balance the concentrations by flowing through the membrane into the higher concentrated solution. This causes an increase in hydrostatic pressure in the higher concentrated solution. If the pressure difference $p_{2}-p_{1}$ equals the osmotic pressure Π, no further diffusion of the solvent takes place [6, p. 206].

#### 2.1.1 Chemical potential

The driving force for a component *j* of a mixture to permeate a membrane is generally the chemical potential $\mu_{j}$ on both sides of the membrane. The chemical potential $\mu_{j}$ of components in a liquid *j* is defined as follows:

(1) $$\mu_{j}\left(T,p,a_{j}\right)=\mu_{j}^{0}+R\;T\ln a_{j}+{\overset{˜}{V}}_{j}\left(p-p^{0}\right)$$

$\mu_{j}^{0}$ is the chemical potential at standard pressure $p^{0}$. The second term on the right side of the equation takes into account the concentration dependence of the chemical potential, while the third term covers the pressure dependency [7, p. 306].

#### 2.1.2 Water activity

In reverse osmosis processes the solvent of interest is water, therefore the water activity $a_{w}$ is described.

According to Scott the water activity $a_{w}$ is a fundamental property of aqueous solutions and can be defined as follows:

(2) $$a_{w}=\frac{p_{w}}{p_{w}^{0}}$$

This expression is valid for isothermal conditions, where $p_{w}$ is the equilibrium partial water vapour pressure of the solution and $p_{w}^{0}$ is the equilibrium vapour pressure of pure water [8, p. 86].

The water activity can also be expressed with the mole fraction $x_{w}$ and the water activity coefficient $\gamma_{w}$.

(3) $$a_{w}=x_{w}\;\gamma_{w}$$

The values of the water activity coefficient $\gamma_{w}$ are in the range of $0<\gamma_{w}<1$, where $\gamma_{w}=1$ represents an ideal solution.

#### 2.1.3 Osmotic pressure

The osmotic pressure Π is the pressure difference $p_{2}-p_{1}$ of the liquids when both sides of the membrane are in equilibrium. Equilibrium is reached when the chemical potentials, as described in Equation (1), are equal.

With pure water on one side of the membrane this results in the following equation [7, p. 309]:

(4) $$\Pi =p_{2}-p_{1}=-\frac{R\;T}{{\overset{˜}{V}}_{j}}\ln a_{j}=-\frac{R\;T}{{\overset{˜}{V}}_{j}}\ln x_{j}\;\gamma_{j}$$

#### 2.1.4 Reverse osmosis

If the solvents are to be moved from the concentrated solution through the membrane into the less concentrated solution, this can be achieved by applying a pressure that is greater than the osmotic pressure Π. This possibility is used in practice e.g. for drinking water production by reverse osmosis [9, pp. 287-288].

### 2.2 Membranes

Membranes are normally flat, partially permeable structures, i.e. structures that are permeable for at least one component of a fluid in contact with them, but impermeable for others.

The flux of component *i* through the membrane can be described as follows:

(5) $$J_{i}=A_{memb}\;\left(\Delta p-\Pi \right)$$

In the previous equation $A_{memb}$ represents the component permeability of the membrane which is a membrane dependent constant, Δ*p* is the transmembrane pressure difference and Π the osmotic pressure according to Equation (4) [5, p. 193].

## 3 Absorbents

### 3.1 Requirements

The two processes absorption and reverse osmosis have different requirements for the absorbent to be used.

On the one hand, a low vapour pressure of the absorbent is important so enough water vapour from the air can be absorbed with a sufficient driving force, and on the other hand, the mass fraction of the solute should be small enough to have a low osmotic pressure.

However, this is a conflict of interest, because the higher the mass fraction of the solute, the better the water vapour can be absorbed from the air, and the lower, the better the water can be recovered by reverse osmosis.

### 3.2 Boundary conditions

To assess the suitability of the absorbents, four different locations for the AWG have been chosen, two of them with extreme conditions in terms of temperature and partial vapour pressure of water in the air (Tamanrasset, Algeria and Abu Dhabi, United Arab Emirates) and the other two with conditions in between those extremes (Polokwane, South Africa and Beitbridge, Zimbabwe).

Based on these locations, values between 10 and 35 °C are used for the ambient air temperature and values between 500 and 3000 Pa for the partial vapour pressure of water in the air.

The values were retrieved from the software Meteonorm [10]. The software is a global climatological database. It enables both the calculation of long-term and current monthly average values as well as high resolution time series of typical years. In this case the monthly averages of typical years were used.

### 3.3 Absorbents

In the course of this work a large number of possible absorbents were investigated. Absorbents which pose a major health hazard, are flammable or unstable were not considered at all.

It has been shown that especially the aqueous salt solutions with lithium bromide (LiBr), lithium chloride (LiCl), potassium hydroxide (KOH) and sodium hydroxide (NaOH) proved to be suitable and have therefore been investigated more closely.

**Figure 1:**
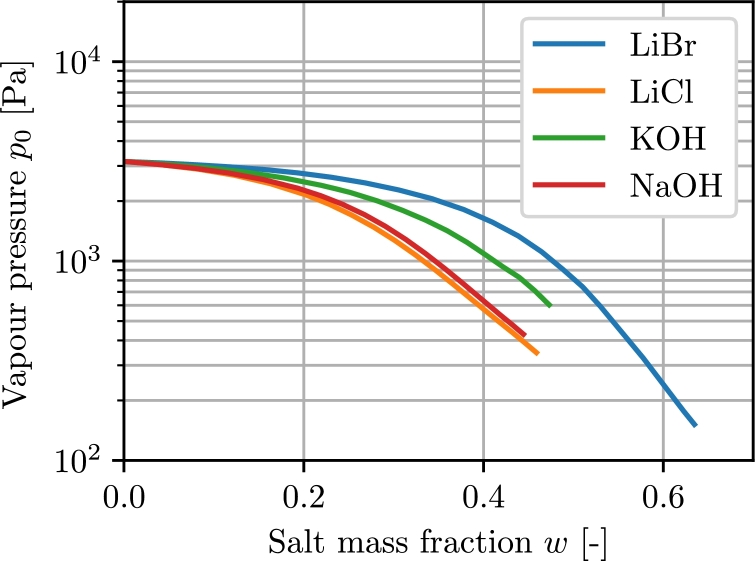
Vapour pressures for selected absorbents at 25 ^∘^C. Derived from [11, pp. 395-396].

As stated above, the vapour pressure of the solution and the osmotic pressure are important material properties to determine the suitability of an absorbent. Fig. 1 shows the vapour pressure of the aforementioned absorbents in dependence of the salt mass fraction.

The end of the respective lines indicates the highest possible salt mass fraction at which the solution is still in the liquid state. At that point the lowest possible vapour pressure can be realised for the given temperature of 25 ^∘^C.

**Figure 2:**
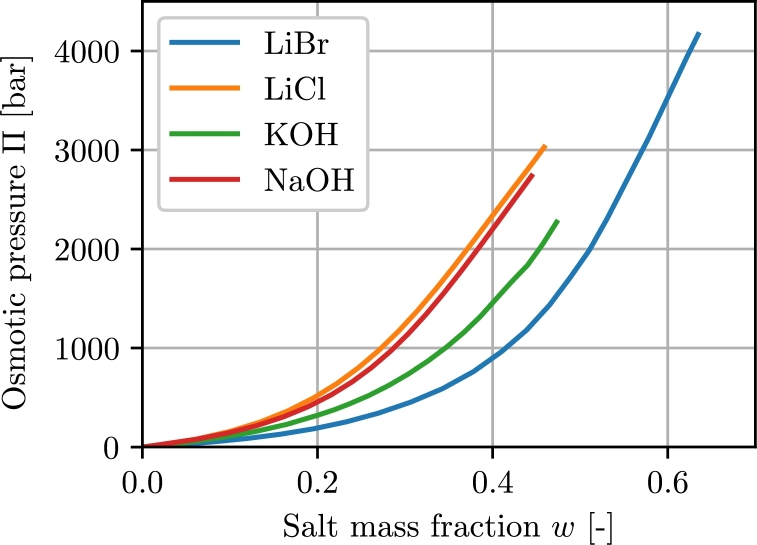
Osmotic pressures for selected absorbents at 25 ^∘^C. Derived from [11, pp. 395-396].

In Fig. 2 the osmotic pressure in dependence of the salt mass fraction is shown.

### 3.4 Selection of absorbent

The investigations showed that the aqueous lithium bromide solution is the most suitable of the above-mentioned absorbents, as it has the lowest toxicity values and low water vapour partial pressures [12], [13], [14].

## 4 Conceptual design

### 4.1 Conventional reverse osmosis

As already seen in the previous chapter, the salt mass fractions will be in the range of about w≈0.4...0.6. This means that osmotic pressures of up to 3500 bar will be exerted across the membrane (Fig. 2). Today's reverse osmosis modules can usually be operated with a maximal pressure of 120 bar [15]. With these 120 bar only about w≈0.1 can be extracted with such a module.

Even if it were possible to develop a module that could be operated at up to 3500 bar, the energy required for pumping would be extremely high and not economical.

For this an alternative was sought, which is explained in the next subchapter.

### 4.2 Multi-stage reverse osmosis

Conventional reverse osmosis modules have no inlet on the permeate side, for high feed salt mass fractions this leads to high osmotic pressures.

**Figure 3:**
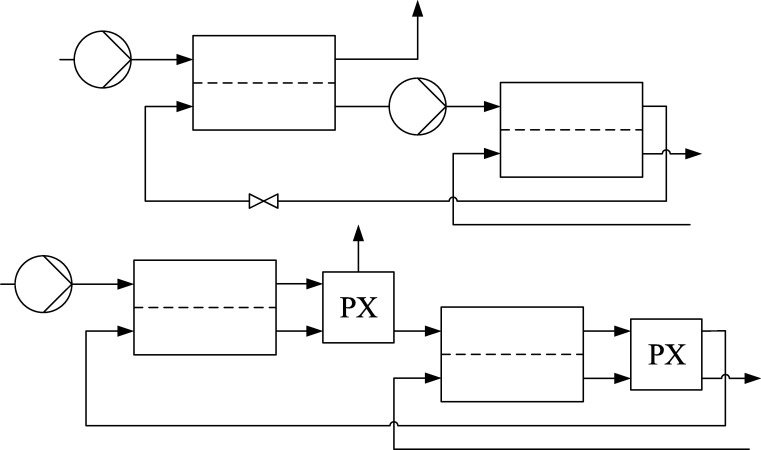
Top: Multi-stage reverse osmosis. Bottom: Multi-stage reverse osmosis with pressure exchanger.

To reduce those osmotic pressures, the module has to be modified with an inlet on the permeate side (Fig. 3 Top).

If a solution with a lower salt mass fraction is now introduced into the permeate side, the concentration gradient and thus the osmotic pressure is reduced.

With this modification the following extended equation for the osmotic pressure can be derived from the chemical potential equation (1), which takes into account the solutes on the permeate side.

(6) $$\Pi =p_{2}-p_{1}=-\frac{R\;T}{{\overset{˜}{V}}_{j}}\ln\frac{a_{j,2}}{a_{j,1}}=-\frac{R\;T}{{\overset{˜}{V}}_{j}}\ln\frac{x_{j,2}\;\gamma_{j,2}}{x_{j,1}\;\gamma_{j,1}}$$

By this modification, the osmotic pressure is distributed over several modules, thus reducing the pressure to be applied per module, but the pressure that has to be applied in total is not reduced.

### 4.3 Multi-stage reverse osmosis with pressure exchanger

Since the total pressure to be applied is not reduced by the modification of the module, a large amount of energy is required for the reverse osmosis process. In order to reduce the required energy, pressure exchangers (PX) are integrated between two adjacent modules (Fig. 3 Bottom). Pressure exchangers transfer pressure energy from a high pressure stream to a low pressure stream. The efficiency is calculated as follows.

(7) $$\eta_{PX}=\frac{\sum {\left(p\;\overset{˙}{V}\right)}_{out}}{\sum {\left(p\;\overset{˙}{V}\right)}_{in}}$$

Pressure exchangers are widely used for seawater reverse osmosis processes and have efficiencies of up to 95% [16].

By using such pressure exchangers, it is no longer necessary to install a pump before each module, it is sufficient to implement a pump once the new feed inlet pressure falls below a certain limit. This results in a massive reduction of the total pressure that has to be applied with the pumps.

**Figure 4:**
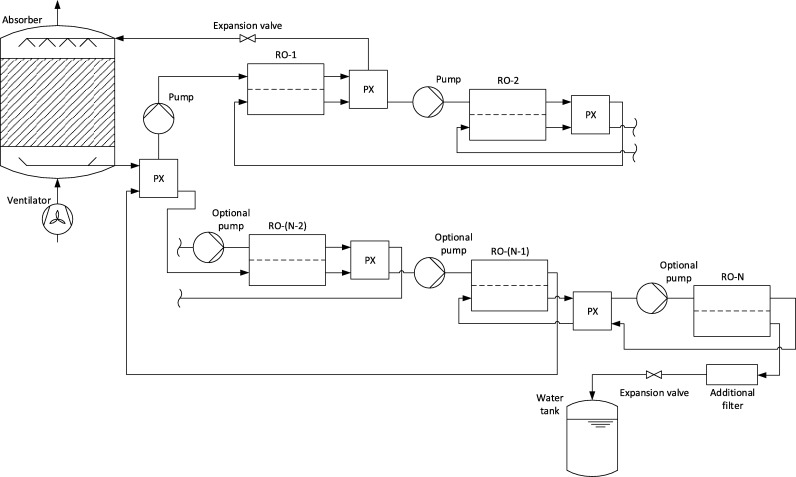
Process schematic for an AWG with absorption and reverse osmosis.

In Fig. 4 the schematic of the whole process for an arbitrary number of reverse osmosis modules is shown. Thereby it can be seen that pressure energy is transferred with a pressure exchanger from the second last to the first reverse osmosis module. This leads to a further significant reduction of the total pressure that has to be applied with the pumps.

The pumps used after the pressure exchangers are booster pumps.

## 5 Modelling

The entire model has been developed in Python [17]. In the models, the thermodynamic properties of the aqueous lithium bromide solution are calculated using Hirschberg's mathematical fits [18, pp. 788-789]. For certain transport properties mathematical fits of an engineering equation solver of the University of Maryland were used [19]. The vapour pressures of water are calculated with the Antoine equation, according to Appendix A.

### 5.1 Absorber

The absorber is modelled as a falling film absorber. Such an absorber has thin plates arranged next to each other along which the solution flows down. From below, air flows into the absorber and flows along the solution up to the top of the absorber. So the air and the solution flow in opposite direction.

#### 5.1.1 Assumptions

The following simplifying assumptions were made to model the absorber:

• The pressure *p* throughout the aqueous lithium bromide solution is constant.
• The liquid film is flat without surface waves.
• The film thickness is considered as constant over the height of the absorber column.
• The inlet solution mass flow rate is considered constant and calculated according to Appendix B.
• The inlet air volume flow rate is considered constant and calculated according to Appendix C.
• The air and solution conditions are constant at a given height in the absorber.
• The influence of non-absorbable gases on the mass transfer is ignored.

For the geometry of the absorber a rectangular cross-section was assumed and a height of 3 m, a width of 2 m and a gap thickness of 0.05 m with 20 gaps was used.

#### 5.1.2 Correlations

According to the *VDI Heat Atlas* the Nusselt number for a plane gap can be calculated with one of the following correlations [20, pp. 785-800].

For laminar flow (Re<2300) the following correlation is used:

(8) Nu1=7.541Nu2=1.841(RePrdh/Lchar)1/3Nu3={21+22Pr}1/6(RePrdh/Lchar)1/2Nu=(Nu13+Nu23+Nu33)1/3

When there is turbulent flow (Re>3⋅104) the following correlation is used:

(9) ζ=(1.8log10⁡Re−1.5)−2Nu=(ζ/8)RePr1+12.7ζ/8(Pr2/3−1)[1+13(dhx)2/3]

$d_{h}$ represents the hydraulic diameter and is for a plane gap defined as $d_{h}=2\;s$, where *s* is the gap thickness.

If the air flow is in the transitional area between laminar and turbulent flow, the Nusselt number gets interpolated linearly.

#### 5.1.3 Calculations

Before the heat and mass transfer calculations are performed in each element, the mass flow rate of dry air must be calculated from the incoming humid air, which is a constant for given boundary conditions.

(10) $${\overset{˙}{m}}_{a,dry}=\left(p_{a}-p_{w}\right)\frac{{\overset{˙}{V}}_{a}}{R_{a}\;T_{a}}$$

$p_{a}$ is the total pressure of the air and $p_{w}$ is the partial water vapour pressure of the air.

Similar to the mass flow rate of dry air, the salt mass flow rate in the absorber remains constant.

(11) $${\overset{˙}{m}}_{LiBr}={\overset{˙}{m}}_{sol,\alpha }\;w_{LiBr,\alpha }$$

From here on, the calculations are performed in each element. First the water mass flow rate from the humid air to the solution is calculated.

(12) $${\overset{˙}{m}}_{w,elem}=\beta_{elem}\;\rho \;A_{elem}\left(X_{w,elem}-X_{w,sol,elem}\right)$$

where $\beta_{elem}$ is the mass transfer coefficient, which can be calculated using the dimensionless Lewis number and is approximately one for air-water vapour mixtures [20, p. 1661].

Then the heat flow,

(13) $${\overset{˙}{Q}}_{elem}=\alpha_{elem}\;A_{elem}\left(T_{a,elem}-T_{w,elem}\right)$$

and the enthalpy flow are calculated. The heat transfer coefficient $\alpha_{elem}$ is calculated using the dimensionless Nusselt number and either Equation 8 or 9.

(14) $${\overset{˙}{H}}_{elem}={\overset{˙}{m}}_{w,elem}\left(\vartheta_{a}\;c_{p,vap}+\Delta h_{v}\right)$$

The heat and enthalpy flow are then used to calculate the enthalpy of the solution in the next element according to the previously mentioned mathematical fits of Hirschberg.

The mass flow rate of the solution increases in each element.

(15) $${\overset{˙}{m}}_{sol,\omega }={\overset{˙}{m}}_{sol,\alpha }+{\overset{˙}{m}}_{w,elem}$$

Therefore the mass fraction changes.

(16) $$w_{LiBr,\omega }=\frac{{\overset{˙}{m}}_{LiBr}}{{\overset{˙}{m}}_{sol,\omega }}$$

With the enthalpy of the solution and the new mass flow rate the specific enthalpy is calculated, which is then needed to calculate the new temperature according to the mathematical fits of Hirschmann.

For the calculations of the air, the water mass flow rate in the air must first be calculated.

(17) $${\overset{˙}{m}}_{w,\alpha }={\overset{˙}{m}}_{a,dry}\;X_{w,\alpha }$$

In contrast to the solution, the water mass flow rate in the air is reduced.

(18) $${\overset{˙}{m}}_{w,\omega }={\overset{˙}{m}}_{w,\alpha }-{\overset{˙}{m}}_{w,elem}$$

The specific enthalpy of the air in the next element is then calculated using the previously calculated heat and enthalpy flows and the dry air mass flow rate.

Then the water load of the next element is determined.

(19) $$X_{w,\omega }=\frac{{\overset{˙}{m}}_{w,\omega }}{{\overset{˙}{m}}_{a,dry}}$$

With the new specific enthalpy and water load of the air, the new air temperature can then be calculated.

#### 5.1.4 Solution algorithm

In the solution algorithm, prior to the calculations of the elements, the boundary conditions are imported. Afterwards the elements of the absorber are calculated from top to bottom. Since the absorber is operated in counter flow and only the inlet values of the air and solution are known, values for the air outlet are assumed. After the subsequent calculation of the absorber, these values are adjusted until the inlet values meet the boundary conditions. Then the solution temperature is adjusted until a stationary state can be reached. These steps are repeated until all criteria are met.

### 5.2 Reverse osmosis membrane module

The reverse osmosis membrane module is modelled as a parallel flow of feed and permeate.

#### 5.2.1 Assumptions

The following simplifying assumptions were made to model the reverse osmosis membrane module:

• There is no temperature change over the membrane.
• The salt rejection of the membrane is 100%, meaning that there is no salt flow through the membrane.

As the model of the reverse osmosis membrane module also involves pumps and pressure exchangers, the following assumptions were made:

• There are no temperature changes over the pressure exchangers or pumps.
• There are no leakages between the streams in the pressure exchangers.
• The ingoing and outgoing volume flow rates are considered constant.

#### 5.2.2 Calculations reverse osmosis membrane

First, the membrane constant is calculated with the water flux determined by the manufacturer under standard conditions [21]. In this model a representative module from DuPont de Nemours, Inc. was used for further modelling [15].

(20) $$A_{memb}=\frac{J_{w}}{\Delta p-\Pi }$$

The following calculations are done in each element. Once the membrane constant is known, the water flow through the membrane can be calculated.

(21) $${\overset{˙}{m}}_{w,elem}=A_{memb}\;A_{elem}\left(\Delta p_{elem}-\Pi_{elem}\right)$$

where $A_{elem}$ is the exchange area of an element, $\Delta p_{elem}$ the transmembrane pressure difference between feed and permeate in an element, and $\Pi_{elem}$ the osmotic pressure according to Equation (6).

Then the mass flow rate of LiBr in the feed, which is considered constant since only water passes through the membrane, is calculated.

(22) $${\overset{˙}{m}}_{LiBr,f}={\overset{˙}{m}}_{f}\;w_{LiBr,f}=\mathrm{const}.$$

Due to the water flowing through the membrane, the feed mass flow rate decreases.

(23) $${\overset{˙}{m}}_{f,\omega }={\overset{˙}{m}}_{f,\alpha }-{\overset{˙}{m}}_{w,elem}$$

The content of LiBr stays constant in the feed, but the water content decreases and therefore the mass fraction of LiBr increases.

(24) $$w_{f,\omega }=\frac{{\overset{˙}{m}}_{LiBr,f}}{{\overset{˙}{m}}_{f,\omega }}$$

Similar to the feed side, the LiBr content on the permeate side is also constant.

(25) $${\overset{˙}{m}}_{LiBr,p}={\overset{˙}{m}}_{p}\;w_{LiBr,p}=\mathrm{const}.$$

As water flows from the feed to the permeate, the mass flow rate of the permeate increases.

(26) $${\overset{˙}{m}}_{p,\omega }={\overset{˙}{m}}_{p,\alpha }+{\overset{˙}{m}}_{w,elem}$$

And therefore the mass fraction of LiBr decreases.

(27) $$w_{p,\omega }=\frac{{\overset{˙}{m}}_{LiBr,p}}{{\overset{˙}{m}}_{p,\omega }}$$

The total mass flow rate through the membrane is the sum of the mass flow rates of the individual elements.

(28) $${\overset{˙}{m}}_{w,tot}=\sum_{i=1}^{N}{\overset{˙}{m}}_{w,elem}$$

#### 5.2.3 Calculations pump and pressure exchanger

The hydraulic power $P_{h}$ of the pump is calculated with the volume flow ${\overset{˙}{V}}_{sol}$ of the solution and the necessary pressure increase Δ*p* [22, p. 48].

(29) $$P_{h}=\overset{˙}{V}\;\Delta p$$

Now the required electrical power $P_{el}$ for the pump can be calculated with the hydraulic power $P_{h}$ and its total efficiency $\eta_{tot}$, which is about 0.8 [23, p. 9].

(30) $$P_{el}=\frac{P_{h}}{\eta }$$

According to Equation (7) the pressure of the next reverse osmosis module $p_{f}$ can be calculated as follows.

(31) $$p_{f}=\eta_{PX}\left(p_{r}+p_{p,\omega }\right)-p_{p,\alpha }$$

where $\eta_{PX}$ is the efficiency of the pressure exchanger, $p_{r}$ the high pressure retentate exiting the previous module, $p_{p,\omega }$ the low pressure permeate exiting the previous module and $p_{p,\alpha }$ the entering permeate from the previous module.

#### 5.2.4 Solution algorithm

In the solution algorithm, prior to calculating the elements, the boundary conditions are imported. Then all elements of a membrane module are calculated from inlet to outlet. Afterwards the inlet permeate salt mass fraction is adjusted until the osmotic pressure is at a level where enough water flows through the membrane. Then a new membrane module is added until the outlet permeate salt mass fraction is below zero. Finally, the last membrane module is recalculated with reduced transmembrane pressure and pure water on the permeate side.

Due to pressure losses, there is a steady decrease in the available feed pressure at the inlet of the new membrane module. To counteract this, a pump is used to increase the pressure whenever it falls below a critical pressure.

### 5.3 System

To estimate the overall performance of the system, the model of the absorber and of the reverse osmosis membrane have been coupled. The model of the system is built according to the schematic in Fig. 4.

#### 5.3.1 Assumptions

The following simplifying assumptions were made to model the entire system:

• Only the steady state is considered.
• There are no leakages between the components.

#### 5.3.2 Solution algorithm

In the solution algorithm the boundary conditions are imported first. Then the absorber is calculated with a certain inlet salt mass fraction. If the target water output is not met, the inlet salt mass fraction is adjusted. Finally, the reverse osmosis membrane modules are calculated with the outlet data of the absorber.

## 6 Simulations

The results of the simulations carried out with the models described in chapter 5 are presented in this chapter.

### 6.1 Input parameters

For the simulations an air volume flow rate of 40'000 m^3^/h, a solution mass flow rate of 1.5 kg/s and a water output of the system of 500 l/d were used as input parameters.

The mass flow rate and the volume flow rate were determined according to the derivations in Appendix B and Appendix C.

### 6.2 Simulation series

According to the boundary conditions of the four different locations various simulations have been carried out. The boundary conditions are based on the meteorological data from chapter 3, so the varying values are the partial pressure of the water vapour in the air $p_{w}$ and the ambient air temperature $T_{air}$.

**Figure 5:**
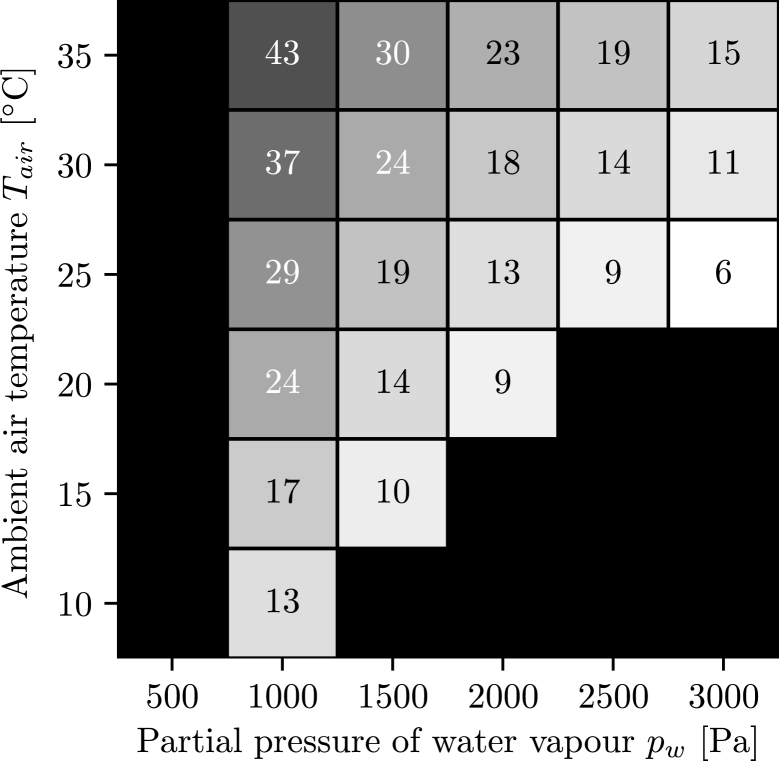
Number of necessary reverse osmosis membrane modules. Black areas left side: Conditions under which, with the chosen absorber dimensions, not enough water can be extracted from the air because the required salt mass fraction is too high and the solution starts to crystallize. (To determine whether the solution begins to crystallize, the solid liquid equilibrium of aqueous lithium bromide is used [24].) Black areas lower right corner: The air is oversaturated, therefore no representative statements can be made.

With the number of reverse osmosis membrane modules, as shown in Fig. 5, statements can be made about the complexity of the entire system. The higher the salt mass fraction, the more stages are required to extract water from it.

**Figure 6:**
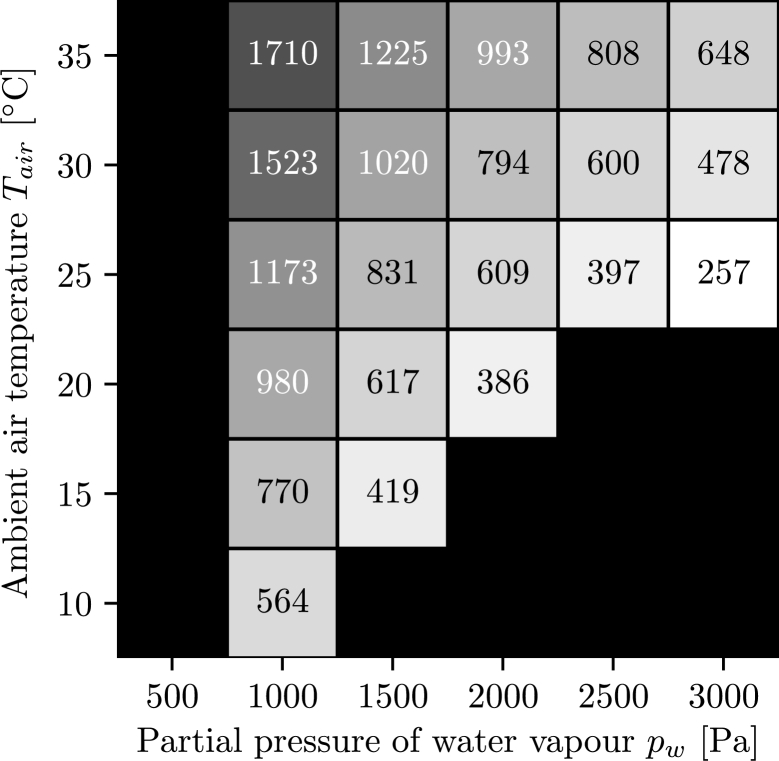
Energy demand per litre [Wh/l]. Black areas left side: Conditions under which, with the chosen absorber dimensions, not enough water can be extracted from the air because the required salt mass fraction is too high and the solution starts to crystallize. (To determine whether the solution begins to crystallize, the solid liquid equilibrium of aqueous lithium bromide is used [24].) Black areas lower right corner: The air is oversaturated, therefore no representative statements can be made.

The energy demand per litre of pure water in Fig. 6 correlates strongly with the number of reverse osmosis membrane modules (Fig. 5), because the pressure losses across the modules and pressure exchangers are constant and therefore the energy required for the pumps to increase the pressures again is similar.

As can be seen in both figures, the number of membrane modules and energy demand decreases with increasing partial pressure of the water vapour or decreasing air temperature.

### 6.3 Exemplary simulation results

Using the simulation data at Tair=20C∘ and $p_{w}=1500$ Pa, the roughly annual mean of Beitbridge and Polokwane, different results of the simulation are shown as examples.

**Figure 7:**
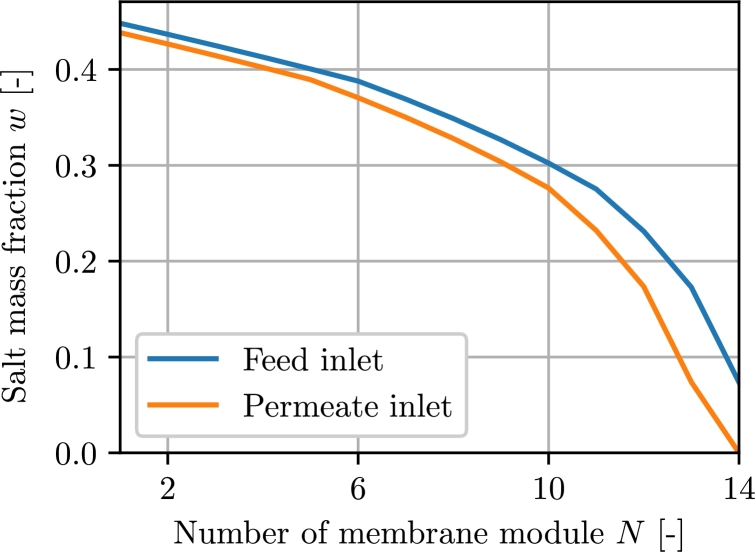
Feed and permeate salt mass fractions at the inlet of the respective membrane modules.

In Fig. 7 the salt mass fractions for the incoming feed and permeate mass flow rates in each module are shown. With decreasing salt mass fraction, the difference between feed and permeate salt mass fraction gradually increases until the permeate salt mass fraction is zero.

**Figure 8:**
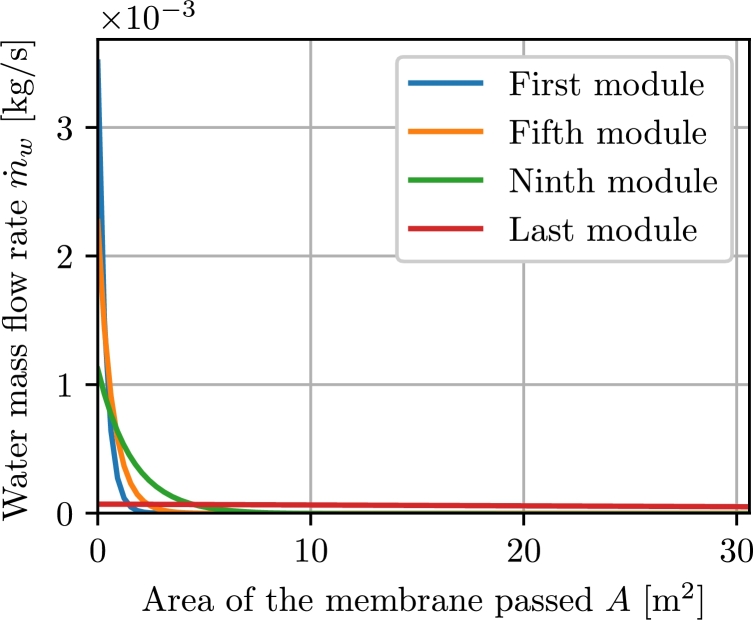
Water mass flow rate through the membrane for selected membrane modules.

The water mass flow rate through the membranes, which is shown in Fig. 8 for four different membrane modules, decreases from the inlet to the outlet. In the first module, most of the water passes through the membrane in the first tenth of the membrane area. As the number of modules increases, the water mass flow rate through the membrane is distributed better until the last module makes use of the entire membrane surface.

Due to pressure losses, the pressure decreases continuously. If the pressure drops below the critical pressure of p=100 bar, it is increased again with a pump. In the last membrane module the osmotic pressure is sufficiently low so that the applied pressure is reduced.

## 7 Discussion and recommendation

In the present paper the water extraction from air with an AWG with absorption and reverse osmosis was investigated.

First, various absorbents were investigated, whereby it was found that especially aqueous salt solutions are suitable. From those, lithium bromide has been chosen for the further course of this work, since low vapour pressures can be achieved and it is the least harmful to health.

A disadvantage, however, is the high salt mass fraction required for the low vapour pressures, because this increases the osmotic pressure and reverse osmosis is no longer possible with conventional membrane modules where there is pure water on the permeate side.

For this reason, a concept was developed which, with a greater technical effort, still makes AWG with absorption and reverse osmosis possible. This concept intends to modify the membrane module with an inlet on the permeate side in order to gradually reduce the salt mass fraction over several membrane modules.

The individual processes absorber and reverse osmosis membrane module were first modelled and then combined into a system.

For the modelling of the reverse osmosis membrane modules the assumption was made that there is no salt flow through the membrane. In reality, however, this is not the case. Although the salt rejection is almost 100%, completely preventing salt from passing through the membrane is not possible. For the model this means that salt, used in the absorber, is continuously removed and therefore has to be added again.

The simulations have shown an energy demand between ∼260–1710 Wh/l. These values start, according to Wahlgren, in the same range as those for condensation processes which require between 270–550 Wh/l [25].

The efficiency of the process could be further improved by using optimized geometries and mass flow rates or by operating the reverse osmosis membrane modules in counter flow.

Most of the modules do not make use of the available membrane area. This is due to the fact that the used membrane module is designed for high water flows and therefore the salt mass fractions in the feed and permeate diverge quickly, resulting in such a high osmotic pressure that no more water passes the membranes.

Generally, this work has shown that an AWG with absorption and reverse osmosis is feasible. For this reason, the operating and investment costs of such an AWG should be calculated. In addition, the effects of small amounts of lithium bromide in drinking water should be studied in more detail. Since there are currently no reverse osmosis membrane modules with a permeate side inlet, the feasibility of such modules should be investigated and then possibly newly developed. As pressure exchangers have a high efficiency for seawater where the salt mass fraction is low, their behaviour at high salt mass fractions should be investigated as well.

**Abbreviations**
AWG: Air water generator/air water generation
PX: Pressure exchanger

**Symbols**
a: Activity [–]
A: Area [m^2^]
Amemb: Membrane constant [kg/(s m^2^ Pa)]
c: Concentration [mol/m^3^]
$c_{p}$: Specific heat capacity (at constant pressure) [J/(kg K)]
d: Diameter [m]
*D*: Diffusion coefficient [m^2^/s]
*g*: Standard gravity [m/s^2^]
h: Specific enthalpy [J/kg]
H˙: Enthalpy flow [W]
J: Mass flux [kg/(s m^2^)]
Lchar: Characteristic length [m]
m˙: Mass flow rate [kg/s]
N: Number of elements [–]
p: Pressure [Pa]
P: Power [W]
Q˙: Heat flow [W]
R: Universal gas constant [J/(mol K)]
$R_{i}$: Specific gas constant of the medium *i* [J/(kg K)]
s: Gap thickness [m]
T: Temperature [K]
v: Velocity [m/s]
V: Volume [m^3^]
V˜: Molar volume [m^3^/mol]
V˙: Volume flow rate [m^3^/s]
$w_{i}$: Mass fraction of component *i* [kg/kg]
x: Position [m]
$x_{i}$: Mole fraction of component *i* [mol/mol]
$X_{i}$: Mass load of component *i* [kg/kg]

**Indices**
a: Air
α: Entering the system
*abs*: Absorption
*avg*: Average
*el*: Electric
elem: Element
f: Feed
*g*: Gas
h: Hydraulic
j: Solvent
l: Liquid
ω: Exiting the system
p: Permeate
r: Retentate
*sol*: Solution
*tot*: Total
*vap*: Vapour
w: Water

**Greek Symbols**
α: Heat transfer coefficient [W/(m^2^ K)]
β: Mass transfer coefficient [m/s]
γ: Activity coefficient [–]
*δ*: Film thickness [m]
η: Efficiency [–]
ϑ: Temperature [^∘^C]
*λ*: Thermal conductivity [W/(m K)]
μ: Chemical potential [J/mol]
*ν*: Kinematic viscosity [m^2^/s]
ρ: Density [kg/m^3^]
Π: Osmotic pressure [Pa]

**Dimensionless Numbers**
$\mathrm{Le}=\lambda /\left(D\;\rho \;c_{p}\right)$: Lewis number
$\mathrm{Nu}=\alpha \;L_{char}/\lambda$: Nusselt number
$\mathrm{Pr}=\nu \;\rho \;c_{p}/\lambda$: Prandtl number
Re=ρvLchar/η: Reynolds number

## Declarations

### Author contribution statement

Marc Fill: Conceived and designed the experiments; Performed the experiments; Analyzed and interpreted the data; Contributed reagents, materials, analysis tools or data; Wrote the paper.

Flavio Muff: Conceived and designed the experiments; Analyzed and interpreted the data.

Mirko Kleingries: Conceived and designed the experiments; Analyzed and interpreted the data; Wrote the paper.

### Funding statement

This work was supported by the Albert Koechlin Stiftung [grant number 19.2.6254].

### Competing interest statement

The authors declare no conflict of interest.

### Additional information

Supplementary content related to this article has been published online at https://doi.org/10.1016/j.heliyon.2020.e05060.

No additional information is available for this paper.

## Supplementary material

The following Supplementary material is associated with this article:

Appendix A.pdf Appendix A - Vapour pressure of water. Appendix A.pdf

Appendix B.pdf Appendix B - Derivation of the solution mass flow rate. Appendix B.pdf

Appendix C.pdf Appendix C - Derivation of the air volume flow rate. Appendix C.pdf

## References

[1] Commission European (Aug. 2010). Water Scarcity and Drought in the European Union.

[2] California Department of Water Resources (2016). Drought Mitigation. https://water.ca.gov/drought.

[3] World Health Organization (WHO) and the United Nations Children's Fund (UNICEF), Progress on Drinking-Water Sanitation, Hygiene: 2017 Update and SDG Baselines (2017).

[4] Shiklomanov I.A. (1993). Water in Crisis: A Guide to the World's Fresh Water Resources.

[5] Baker R.W. (2004). Membrane Technology and Applications.

[6] Kleiber M. (2016). Process Engineering. https://www.ebook.de/de/product/20478219/michael_kleiber_process_engineering.html.

[7] Kraume M. (2012). Transportvorgänge in der Verfahrenstechnik.

[8] Scott W.J. (1957). Water Relations of Food Spoilage Microorganisms.

[9] Baerns M., Behr A., Gmehling J., Hofmann H., Onken U. (2013). https://www.ebook.de/de/product/20503146/manfred_baerns_arno_behr_juergen_gmehling_hanns_hofmann_ulfert_onken_technische_chemie.html.

[10] https://meteonorm.com/.

[11] Barbosa-Cnovas G.V., Fontana A.J., Schmidt S.J., Labuza T.P. (2007). Water Activity in Foods: Fundamentals and Applications.

[12] https://www.dguv.de/ifa/gestis/gestis-stoffdatenbank/index.jsp.

[13] Hui L., Edem N.K., Nolwenn L.P., Lingai L. (2011). Evaluation of a seasonal storage system of solar energy for house heating using different absorption couples. Energy Conversion and Management.

[14] https://www.caymanchem.com/msdss/10005316m.pdf.

[15] DUPONT Dupont (2019). XUS 180808 reverse osmosis element. https://www.dupont.com/content/dam/dupont/amer/us/en/water-solutions/public/documents/en/45-D01736-en.pdf.

[16] Cameron I.B., Clemente R.B. (2008). SWRO with ERI's PX pressure exchanger device — a global survey. Desalination.

[17] (2019). https://www.python.org.

[18] Hirschberg H.G. (1999). Handbuch Verfahrenstechnik und Anlagenbau.

[19] LiBrSSC (aqueous lithium bromide) property routines. http://fchart.com/ees/libr_help/ssclibr.pdf.

[20] VDI-Gesellschaft Verfahrenstechnik und Chemieingenieurwesen (Ed.), VDI-Wärmeatlas, 11th Edition, Springer Berlin Heidelberg, 2013.

[21] Moser M., Micari M., Fuchs B., Farnós J. (2018). Software tool for the simulation of selected brine treatment technologies.

[22] Böswirth L., Bschorer S. (2014). Technische Strömungslehre.

[23] Hatami H. Hydraulische Formelsammlung. http://www.boschrexroth.ch/business_units/bri/de/downloads/hyd_formelsammlung_de.pdf.

[24] Conde-Petit M.R. (2014). Solid - Liquid Equilibria (SLE) and Vapour - Liquid Equilibria (VLE) of Aqueous LiBr.

[25] Wahlgren R.V. (2001). Atmospheric water vapour processor designs for potable water production: a review. Water Res..

