# Supplementary material for: Evaluation of a new air water generator based on absorption and reverse osmosis
Source: Heliyon. 2020 Sep 25;6(9):e05060. doi: 10.1016/j.heliyon.2020.e05060 (PMC7522095; doi:10.1016/j.heliyon.2020.e05060)
Supplement: Appendix A.pdf — Appendix A - Vapour pressure of water. [file mmc1.pdf]

Appendix A - Vapour pressure of water

The vapour pressure of water, in kPa, is calculated with the Antoine equation,

$$\log p_0 = A - \frac{B}{C + \vartheta} \tag{1}$$

where A, B and C are the Antoine coefficients and ϑ is the temperature in °C.

Between 1 and 100 °C the Antoine coefficients $A = 7.19621$, $B = 1730.63$ and $C = 233.426$ can be used [1, p. 439].

References

[1] S. Rönsch, Anlagenbilanzierung in der Energietechnik, Springer Fachmedien Wiesbaden, 2015. doi:10.1007/978-3-658-07824-9.
