# Supplementary material for: Evaluation of a new air water generator based on absorption and reverse osmosis
Source: Heliyon. 2020 Sep 25;6(9):e05060. doi: 10.1016/j.heliyon.2020.e05060 (PMC7522095; doi:10.1016/j.heliyon.2020.e05060)
Supplement: Appendix B.pdf — Appendix B - Derivation of the solution mass flow rate. [file mmc2.pdf]

Appendix B - Derivation of the solution mass flow rate

To estimate the mass flow rate of the LiBr-H_2O solution entering the absorber, an average velocity of the fluid film must be determined.

According to Nusselt's film theory this can be done with the following equation.

$$v_{avg} = \frac{(\rho_l - \rho_g)\, g}{3\, \eta_l} \delta^2 \tag{1}$$

The average velocity v_{avg} is therefore dependent on the film thickness δ, the standard gravity g and the properties of the liquid and the surrounding gas, such as density and viscosity [1, p. 482].

Most of these values are known, except the film thickness. According to Zhang et al. the film thickness for LiBr-H_2O solutions with laminar flow is between 0.2 and 0.5 mm [2]. Inserting these film thicknesses and the material properties (for LiBr-H_2O solution with $w = 0.5$ at 20 °C) in Equation 1 results in an average flow velocity v_{avg} range of 0.05 to 0.3 m/s.

Together with the number of gaps in the absorber ($N = 20$), the depth of the absorber ($depth = 2$ m) and the film thicknesses, a range of volume flow rates can be calculated. These can then be converted easily to mass flow rates between 1.2 and 18.2 kg/s.

In order to compare the results of the different simulations, the same mass flow rate of 1.5 kg/s has been used for all of them. A mass flow rate at the lower limit was chosen because the energy demand is directly proportional to it.

References

[1] H. D. Baehr, K. Stephan, Wärme- und Stoffübertragung, Springer Berlin Heidelberg, 2010. doi:10.1007/978-3-642-10194-6.

[2] L. Zhang, Y. Wang, Y. Fu, L. Xing, L. Jin, Numerical Simulation of H_2O/LiBr Falling Film Absorption Process, Energy Procedia 75 (2015) 3119–3126. doi:10.1016/j.egypro.2015.07.644.
