# Supplementary material for: Evaluation of a new air water generator based on absorption and reverse osmosis
Source: Heliyon. 2020 Sep 25;6(9):e05060. doi: 10.1016/j.heliyon.2020.e05060 (PMC7522095; doi:10.1016/j.heliyon.2020.e05060)
Supplement: Appendix C.pdf — Appendix C - Derivation of the air volume flow rate. [file mmc3.pdf]

Appendix C - Derivation of the air volume flow rate

In order to determine the volume flow rate through the absorber, a suitable ventilator had to be identified. Therefore, a simple ventilator that consumes relatively little energy was searched for in a manufacturer's catalogue. Such a ventilator was found in a catalogue by ZIEHL-ABEGG [1, p. 134].

Said ventilator can be operated at up to 40000 m^3/h where it needs an input power of 880 W.

References

[1] ZIEHL-ABEGG, Axialventilatoren Hauptkatalog.
URL https://www.ziehl-abegg.com/fileadmin/Downloadcenter_NEW/02_deutsch/X02_Catalogues/Katalog-Axialventilatoren-Hauptkatalog.pdf
